# A pilot study of the individual placement and support model for patients with chronic pain

**DOI:** 10.1186/s12891-017-1908-3

**Published:** 2017-12-28

**Authors:** L. Rødevand, T. M. Ljosaa, L. P. Granan, T. Knutzen, H. B. Jacobsen, S. E. Reme

**Affiliations:** 1 Department of Pain Management and Research, Oslo University Hospital, Oslo, Norway; 2 Department of Psychology, Faculty of Social Sciences, University of Oslo, Postboks 1094 Blindern, 0317 Oslo, Norway

**Keywords:** Individual placement and support, IPS, supported employment, Chronic pain, Pain management, Rehabilitation medicine, Work disability, Interdisciplinary, Integrated care, Coping

## Abstract

**Background:**

Individual Placement and Support (IPS) is an evidence-based work rehabilitation model with well-documented effects for people with mental illness. The model has, however, never been tested out for people with chronic pain. This pilot study aimed to investigate chronic pain patients’ experiences with the IPS job support model.

**Methods:**

We recruited eight consecutive patients referred for various chronic pain conditions at a hospital outpatient pain clinic. They were offered IPS job support as an integrated part of their interdisciplinary pain rehabilitation. The patients’ experiences were investigated through semi-structured interviews 3 months after inclusion in the study.

**Results:**

The participants reported mostly positive experiences with IPS. One participant dropped out of the study after deterioration of symptoms, while the remaining participants were satisfied with the intervention. Particular helpful aspects of the IPS intervention were the follow-up from the employment specialist, focus on competitive employment, focus on work despite pain complaints, reframing work into something positive, administrative support, and practice in writing applications. No participants reported adverse experiences from the IPS intervention. Within a 12-months time frame, 3 of the 8 participants gained competitive employment.

**Conclusions:**

This is the first report of the IPS model of supported employment applied in an outpatient setting for chronic pain patients. The results suggest that IPS can be successfully integrated with interdisciplinary pain rehabilitation, and warrants large-scale testing in a randomized controlled trial.

## Background

Chronic pain prevalence is between 20% and 30% in the Norwegian adult population [1, 2]. A common consequence of persistent pain is reduced work capacity and exclusion from the labor marked [3]. The socioeconomic costs are vast. Long-term back pain alone costs annually more than 4 billion euros in sick leave, and more than 6 billion euros in social welfare in Norway [4]. Long-term sick leave is also associated with individual consequences such as reduced quality of life [5, 6].

There is international consensus on the need for a multidisciplinary approach to complex pain conditions where no single medical treatment suffice [7]. Moreover, evidence suggests that an integrated approach in treatment is better than a sequential process [8]. Within an integrated approach, health care professionals with different educational backgrounds have complementary roles and work together to assess and treat the patient. For example, a patient with persistent pain may need medication to dampen symptoms, physical training to strengthen muscles and balance, cognitive and emotional treatment to increase function and quality of life, and support to manage work. Work-related follow-up is however rarely provided in pain clinics, despite the recent focus on integrating work and health in all patient treatments [9].

Vocational rehabilitation and training approaches generally fall within two traditions, train and place or place and train. In the former approach, the client undergoes targeted training in an adapted or sheltered environment to acquire necessary skills before attempting to enter competitive employment in an arena relevant to the training. In contrast, the place and train principle represents a novel approach where the primary goal is direct employment in real-life settings, without any preceding training. Individual Placement and Support (IPS) is the evidence-based model within this tradition, where the goal is to provide individual job support to help people with disabilities participate in the competitive labor market [10]. IPS was originally developed to help people with mental disorders to obtain and maintain employment [11, 12], and integrates job support as a component of the psychological treatment rather than a separate service. It focuses on the patients’ preferences, with the philosophy that anyone who wants to work can find a regular job in the community, and that no one should be deprived of that opportunity [13]. Within the IPS model, patients are not screened for work readiness, but rather on expressed desire to work. It does not involve intermediate work experiences, transitional employment or sheltered workshops, but actively facilitates job acquisition and provides ongoing support once the patient is employed. International research shows that IPS is substantially more effective than other types of employment programs for this group of patients [14, 15], and has also demonstrated improvements in non-vocational outcomes, such as better symptom management and better self-esteem [16]. Although the effectiveness of IPS for patients with mental illness is well documented, IPS has previously not been investigated in patients with chronic pain, despite an overlap between the two conditions [17–20]. Indeed, pain itself is defined as consisting of affective and motivational as well as sensory components [21, 22].

The aim of this pilot study was to investigate chronic pain patients’ experiences with the IPS job support model as an integrated part of the interdisciplinary pain treatment in an outpatient hospital pain clinic.

## Methods

### Recruitment

Eight patients from a pain clinic in a major hospital in the Oslo area were recruited to the study by their treatment provider (physician, psychologist or physiotherapist). In the recruitment period, eight consecutive patients were invited during their consultation at the clinic. None of the invited patients declined to participate, and all of the patients signed the informed consent. Inclusion criteria involved (1) expressed motivation to work, (2) not currently working (long-term sick leave, disability pension or unemployed), and (3) eligible for interdisciplinary pain treatment. Eligibility for interdisciplinary pain treatment is evaluated by an interdisciplinary admission team at the clinic based on the information provided in the patient’s referral: Pain condition (duration, severity, impact on daily life), prior examinations and treatments, current medications, and comorbidities. In terms of the sample size, we considered eight participants to be sufficient to be able to respond to the study aim. The study was approved by the Data Protection Officer at Oslo University Hospital.

### Intervention

The IPS job support is delivered by employment specialists adhering to a detailed manual [23]. IPS incorporates the following eight principles in the approach to vocational rehabilitation: (1) eligibility based on the patient’s own choice, (2) focus on competitive employment (i.e., jobs in integrated work settings in the competitive job market at prevailing wages with supervision provided by personnel employed by the business), (3) integration of mental health and employment services, (4) attention to patients’ preferences, (5) work incentives planning, (6) rapid job search, (7) systematic job development, and (8) individualized job support [24]. This implies that the job search and support are adapted to the individual’s needs and challenges. The eight IPS principles were adhered to in the current study, except for a small modification to the third principle. The employment services were not integrated with mental health treatment, but with interdisciplinary pain treatment.

All participants received IPS in addition to treatment as usual. An employment specialist with long experience in IPS (TK) provided job support according to the 8 IPS principles. Usual treatment at the pain clinic involves interdisciplinary pain management provided by one or two physicians (anesthesiologist, gynecologist, neurologist or specialist in physical medicine and rehabilitation), psychologists, physiotherapists and nurses. At least two professions followed the participants up over a time-period of one to 12 months, with a frequency of every other week to once a month. The employment specialist and the pain management team had weekly meetings where they discussed all participants. Usual care within vocational rehabilitation in Norway usually involves train and place approaches such as Work with assistance and Traineeship in a sheltered business. This is also what pain patients on long-term disability are offered from the public welfare services, in addition to their medical treatment, with little or no integration between the vocational rehabilitation and medical treatment.

Originally, the pilot study aimed to provide IPS job support in addition to usual treatment for 3 months only. However, since an opportunity to follow some of the participants unfolded upon completion of the 3-months, we decided to offer continuing job support to the four participants in most need of it. Four participants were thus followed-up for additional 9 months by an employment specialist, while the remaining four participants were followed up by clinicians only (see Table 2).

### Study context

The Norwegian public insurance system includes all lawful residents of Norway and provides health service benefits and pensions for all members of the National Insurance Scheme, administered by the Norwegian Welfare and Labor Administration. The workers’ compensation program is part of the scheme and provides 100% coverage for lost income due to medically acknowledged sickness, disease or injury from day one until the person can work again, up to 52 weeks. After that, long-term benefits provide approximately 66% of former income. All participants in the pilot study received long-term benefits due to their pain condition.

### Instruments and measurements

Demographical and clinical characteristics were obtained from a local pain registry. This local registry is already established in the pain clinic, and a standardized screening of all patients is conducted before any clinical consultation. The following registry data was obtained in order to describe this study sample: modified Oswestry Disability Index (ODI)^1^ [25] to provide a measure of disability, Numeric Rating Scale to assess pain intensity and pain bothersomeness, The Hopkins Symptom Checklist (HSCL-25) [26] as a measure of psychological distress, and European Quality of Life**-**5 Dimensions (EQ-5D) [27] as a measure of health-related quality of life. Employment status was registered at baseline, 3 months, 6 months and 12 months follow-up, and was registered through telephone conversations or during follow-up consultations.

Participants’ experiences with IPS were investigated through individual semi-structured interviews. The interviews were taped with a digital audio-recorder. One investigator (LR) conducted face-to-face interviews approximately 3 months after IPS had started. Location of the interviews was either at the clinic or in the home of the participants, depending on their preferences. In addition to the open-ended-questions in the interview guide (see below), other questions and prompts were posed if considered necessary to encourage the participants to elaborate on issues spontaneously raised by him or her. During the interviews, general expressions of interest were also used (e.g. “mhm”, “that’s interesting”, “I see”). The interviewer attempted to establish a warm, empathic atmosphere to build rapport. Prior to the interview, the interviewer explained the objectives of the interview to the participant, guaranteed confidentiality, and both agreed on a scheduled appointment for the interview. This procedure may also have helped build rapport. The length of the interviews ranged from 13 to 33 min (M = 22, SD = 9).

#### Questions to all participants

- Tell me about your experiences with the job support as a part of the treatment offered at the pain clinic.
- What do you think about IPS as a part of the treatment at the pain clinic?
- Have you experienced any barriers or difficulties getting started with the work process?

#### Questions to participants who were currently working

- Tell me about your experience of being (back) at work?
- How do you feel now compared to before you received the job support?
- How has work affected your daily life and function (including physical, social and mental function)?
- How has work affected your symptoms?
- Is there anything else you want to add?

#### Questions for participants who were not yet working

- Tell me about your experience with the job search process?
- How do you feel now compared to before you started receiving job support?
- How has participation in this study affected your daily life and function (including physical, social and mental function)?
- Is there anything else you want to add?

### Data analysis

We used the qualitative method of Systematic Text Condensation (STC) as described by Malterud and colleagues [28] in order to analyze the interview data. One investigator (LR) listened to the audiotapes repeatedly and transcribed the audio files into text files. Three investigators (LR, SER, and TML) read the transcripts thoroughly, and investigated for common and salient themes. The three investigators read and analyzed the transcripts separately to widen the analytic space and derive at some consensus, as the STC approach suggests [28]. They identified manifest themes inductively from the data. In contrast to a deductive approach, an inductive approach involves sorting and coding themes that draws on the raw data itself, and not on existing theories [29]. The STC procedure consists of four steps: 1) Get a general impression of the material, and identify themes. 2) Identify and sort meaning units, and sort themes in code groups including meaning units. 3) Perform condensation: Split code groups with meaning units into sub groups. In addition, transform meaning units into a more general format, called a “condensate” (an artificial quotation). 4) Summarize data and develop an analytic text that presents the most salient content and meaning based on the condensates (step 3). Descriptions and concepts are also developed [28].

According to the STC method, the researchers must be aware of their potential influence on the results of the analysis. We therefore reflected upon our interpretations of the interview material, attempted consistently to be loyal to the participants’ experiences, and aimed to depict their story in an honest way. Other studies of pain patients have also used the STC method, thus demonstrating the utility of this strategy for studies within the field of chronic pain [30, 31].

Quantitative data was analyzed with descriptive statistical methods, using SPSS statistics software version 21. The main purpose of the quantitative analyses was to describe the participants’ sociodemographic and clinical characteristics to ensure transferability and allow for triangulation of results.

## Results

### Clinical characteristics

The study population comprised 8 participants, four men and four women. The age of the participants ranged from 22 to 51, and the majority was in their thirties. The pain conditions of the participants included chronic low back pain, chronic neck/back pain, chronic knee pain, chronic abdominal pain, post-stroke pain, elbow- and neck pain, chronic widespread pain, and post-traumatic headache. All participants reported a pain duration of at least 2 years (2–16 years), and none of them had been in competitive employment for the last 2 years or more (2–16 years). For more detailed information on clinical characteristics, see Table 1.

**Table 1:** Clinical characteristics of the participants

Participant	ODI disability	Pain intensity 0–10	Pain bothersomeness 0–10 NRS	Health thermometer (0–100)	HSCL-25 total	HSCL-25 anxiety	HSCL-25 depression
1	52	8	8	28	2,0	1,8	1,6
2	34	8	8	79	2,4	2,6	2,3
3	40	5	7	55	2,5	1,7	3,2
4	–	4	4	–	1,8	2,0	1,6
5	14	8	7	51	1,4	1,3	1,1
6	48	8	10	30	2,7	2,7	2,6
7	70	8	9	4	3,1	2,8	3,7
8	14	5	6	50	1,7	1,3	1,7

*Abbreviations*: *ODI* Oswestry Disability Index, *NRS* Numeric Rating Scale, *HSCL-25* Hopkins Symptom Check List

One participant dropped out of the study a few weeks after enrollment due to deterioration of symptoms. The participant was nevertheless included in the post-intervention interview.

### Employment status

After 3 months follow-up, none of the participants had gained employment. After 6 months, one of the participants had gained competitive employment. At 12 months, another two of the participants had gained employment, leaving an employment rate of 38% over the study period of 12 months (Table 2).

**Table 2:** Date of IPS inclusion, IPS termination, job start and duration of IPS

Participant	IPS inclusion date	IPS termination date	Job start date	Duration of IPS (in months)^a^
1	17.04.2015	08.05.2015	–	0.7
2	24.04.2015	17.07.2015	09.09.2015	4.6
3	27.04.2015	17.04.2016	11.01.2016	8.6
4	11.05.2015	17.04.2016	25.04.2016	9.5*
5	03.06.2015	17.07.2015	–	0.8*
6	09.06.2015	17.04.2015	–	9.2*
7	10.06.2015	17.04.2016	–	9.4*
8	17.06.2015	17.07.2015	–	1.0

*Abbreviations*: *IPS* Individual Placement and Support

^a^Not including time spent on holiday, waiting for surgery, or being hospitalized

### Results from semi-structured interviews

We identified four main themes from the interviews: (1) the overall experience of IPS; (2) perception of IPS as part of the pain treatment; (3) impact of IPS and (4) experience of the job search process. The result section is organized according to these four themes. Quotes are used to exemplify and illustrate the themes. The quotes are condensates of the content, and aimed at maintaining the original terminology used by the participants.

1. **The overall experience of IPS**

This theme describes the participants’ overall experiences with IPS, and outlines the IPS elements that the participants found particularly helpful in the job support process.

All participants, apart from the drop-out, reported positive experiences with the IPS. Some participants highlighted that the regular follow-up and encouragement from the job specialist was helpful: “It is a good feeling to be followed-up and get the extra attention ... An attempt to be pushed in a direction, a positive direction ... To hear: *You can do this.*” (P3). One participant described the consistent work focus as helpful: “It has helped me to think a bit more about activity and work as part of the pain rehabilitation process.” (P4). The same participant also said that he had realized that he copes better with certain activities. For example, administrative tasks made him tired, whereas rock climbing had a positive impact on him: “Earlier, I focused on regulating the amount of activity. Then the doctor suggested: *Let’s focus on rock climbing*. That made me think; maybe I shouldn’t seek a certain amount of activity, but rather a certain type of activity.”

Another participant described that the IPS element of work despite pain complaints was particularly helpful (P7). Others highlighted that the study reframed work into something positive, and that this focus “started a thought process”, as one participant (P4) stated. This participant also claimed that the support in job seeking activities was very helpful, such as booking meetings with employers. Other participants emphasized that practicing writing applications was important to them: “I am now better at writing, formulating and getting my message across.” (P3).

No participants described adverse events from the IPS. However, one participant reported a negative experience with IPS. She dropped out early of the study after experiencing more pain and fatigue: “We (she and the job specialist) spoke about many things, and I just became tired of thinking.” (P1).

(2) **Perception of IPS as part of the pain treatment**

This theme describes the participants’ thoughts and expectations about IPS as an integrated part of the treatment at the outpatient pain clinic.

Several participants highlighted the significance of work for mental and physical well-being. “I think it is a very good, a very smart program ... I think it can work well for many people. Because you become ill of being ill ... Not just physically, but mentally as well ... You get a little stuck.” (P8). Another participant was convinced that long-lasting job support, like IPS, could initiate a positive process that contributes to employment with beneficial effects on health. The alternative, unemployment, “is the root of all evil … It is not beneficial when it comes to illness ... You get low self-esteem ... You don’t contribute to anything ... You start to doubt your abilities, feel like a burden to the society, and get no sense of achievement.” (P2). The same participant highlighted that an important consequence of work is that you feel useful: “I think every human being, despite challenges, will benefit from work and will feel useful in one way or another … Unfortunately, though, the workplace is not adapted to everyone, and it can’t be … because there are so many individual needs” (P2). She pointed out that IPS, on the other hand, helps people find a job that is suitable to the individual needs and preferences.

Some participants expected that work would be an opportunity to get distraction from the pain: “I think it is better to work instead of sitting at home and just wait for the next pain episode.” (P2).

All participants, except for the drop-out (P1), claimed that IPS did not interfere with the treatment they received at the pain clinic. While the majority spoke in favor of including IPS as part of the pain treatment, the drop-out would rather postpone the job support until after she had finished her physiotherapy treatment.

(3) **Impact of IPS**

This theme describes the impact of IPS on the participants’ mental, social and physical function.

#### Mental function

All participants, except from the drop-out (P1), reported a positive impact of IPS on their mental state. The majority expressed a stronger hope: For many participants, hope seemed to be connected to a feeling of moving in a positive direction: “It has been a feeling of getting started … Maybe a feeling that I am heading somewhere … out of a fixed state” (P2). Hope was also expressed by a change in mind set: “Even if it (IPS) hasn’t resulted in a job, it probably has done something to the whole mind set … To think about something else and think that life pretty soon may be different.” (P2). Or as one participant put it: “I see a way forward” (P4).

One participant described an immediate increase in positive affect after being introduced to IPS: “I felt happy … that I was on the way somewhere else than the living room.” (P8). The same participant also reported that the “spark” and dreams had returned: “It (IPS) has given a little spark … You lose some of that spark when you face permanent disability pension at a young age … Now, the dreams are coming back, which is very positive, mentally speaking.”

However, one participant described an immediate concern about how to manage work when in pain: “I am a little afraid of how my medical state will respond to work. Occasionally, when I experience intense pain or other distress associated with the disease, I think: Oh my god, this will never work … This kind of reasoning is a little more frequent … but only now and then.” (P2). The same participant was also concerned about how to cope socially at work: “If I focus too much on the pain, I can’t focus on the conversation … And then I start to think: Oh my god, did I say that? or Did I stand there and act weird? ... I kind of become a little neurotic.”

#### Social function

Most of the participants reported that the IPS job support had had no impact on their social function. However, a few of the participants expressed a change in the social domain. One participant said that she participated more readily in conversations. “I feel that I now have something to talk about … On the next birthday party or the next meeting, maybe I actually have something to talk about apart from the boring … You probably don’t notice it because you’re well, but there are incredibly many conversations where it doesn’t take more than 20 minutes until the subject is work.” (P8). Another participant described how embarrassing it was to reveal the medical condition to the job specialist. Although embarrassing, this revelation was at the same time a “kind of a barrier to cross.” (P2).

#### Physical function

Some participants described a physical improvement. For example, a participant started to work as a rock climbing instructor during the study, and expressed a significant improvement: “It is interesting because when I’m out climbing, I’m relaxed and feel no pain ... When I’m physically active ... not focusing on myself so much ... and have that instructor role ... I feel extremely well ... I feel almost like before.” (P4). Another participant (P2) also experienced physical improvements during the study. Whether the improvements could be attributed to IPS or not was however uncertain.

#### Negative impact

None of the participants experienced a negative impact of IPS on their lives. However, the drop-out (P1) described an immediate increase in pain and fatigue when she started IPS and physical therapy at the same time. She further attributed the simultaneous involvement in the two interventions to have caused the exacerbation of symptoms.

(4) **Experience of the job search process**

This theme describes the participants’ experience of the job search process, including possibilities, positive aspects as well as challenges.

#### Possibilities and positive aspects

Seven of eight participants described the job search process as a positive experience. For example, one participant noted that “It is a very exciting process and I am, like, very positive to it.” (P3). Another participant expressed more hope about work prospects and the future: “That experience gave me hope ... And just to come to an interview … is a positive thing.” (P7). One participant compared the job search process to “selling” and something enjoyable: “For me, job searching is amazingly interesting … It is like selling. You kind of sell yourself.” (P4).

One participant described a desired change in life, and went from applying for permanent disability pension to applying for a job instead: “It is something I am very happy about … I was about to be granted permanent disability benefit. I was actually pretty far into that process … When that process stopped, and I now have the opportunity to work and be … “normal” … that’s very, very positive. It gives you a good feeling.” The participant elaborated on that feeling: “It is a good feeling to actually say that I’m applying for a job when someone asks what I am doing … To say something else than that I am sick.” (P8).

#### Challenges

The participants experienced some challenges during the job search process, such as their lack of education, a previous history of long-term work absence, the writing of applications, gaps on the CV, pain, fear of setbacks, and short duration of the pilot study.

All except from one of the participants reported limited or no education as a challenge. Some described education as the main obstacle: “My biggest problem is that I have no education.” (P8). Others stated that they did not have enough, or the right type of education, for the jobs that they found interesting: “It is challenging to find a job that is interesting and that doesn’t demand a specific education.” (P2).

Some participants pointed out that long-term work absence was another challenge: “What’s most difficult is that I have been outside the labor market … It feels so hard to pick up where I left off as a 21 year old, now that I’m in my late thirties. To sit in a reception … or do another job that I think a monkey could do … I think that’s sad … I have higher ambitions for myself.” (P2). Another participant further described that the history of long-term work absence had a negative impact on the self-esteem. He reported that he had started to doubt himself and his abilities: “My whole life has become a disease … Of course it breaks me down when it comes to self-esteem … I ask myself: What can I do at all?” (P7).

One participant in particular highlighted difficulties with writing applications as a major obstacle: “The challenge has been that I, for the first time in my life, have written an application and CV.” (P3). Note that some of the participants actually reported writing applications to be one of the helpful elements of the IPS (see above). Gaps on the CV was also described as a challenge by some of the participants: “When I was writing my CV … I lied … to fill in the gaps … I revised it so that it wouldn’t look like I had a gigantic 16 years gap on the CV.” (P2).

Several participants focused on pain as the main challenge in the job search process: “It’s the pain that’s the challenge ... Because I don’t know whether I will be able to work full time … You don’t know how long it will take until you’re better.” (P3). Another participant expressed a discrepancy between what she wanted and what her body could manage: “The motivation ... is there. The only thing that is lacking is the cooperation ... from my body. It doesn’t cooperate.” (P1).

Several participants expressed a fear of setbacks if they started working: “I’m a bit anxious … What if I get a job and then become ill … Then, maybe I lose the job.” (P8). She feared that she would not be able to manage the job and thus experience a “new defeat … that is the biggest challenge, not managing it.” Other participants expressed a similar worry related to their work capacity and health: “How long will it take until I am on sick leave again?” (P6).

Six of the eight participants considered the short duration of the job support to be a challenge. They would have ideally wanted a longer duration of the job support, and one participant in particular emphasized that this was the reason why he did not manage to return to work (P2).

## Discussion

The primary aim of this pilot study was to investigate chronic pain patients’ experiences with the IPS job support model delivered as part of the interdisciplinary pain treatment. The semi-structured interviews showed mostly positive experiences as reported by the participants. Particular helpful aspects seemed to be; a) follow-up and encouragement from the employment specialist, b) focus on competitive employment, c) focus on work despite pain complaints, d) reframing work into something positive, d) administrative support and e) practice in writing applications. What these findings highlight in particular is the necessity of providing individualized job support where the focus is on competitive employment as opposed to work rehabilitation in sheltered settings. The participants highlighted the value in getting help to find a job that was suitable to their individual needs and preferences, not just any job. Experiences during the job search process, e.g. coming to a job interview or being in contact with potential employers, further provided them with encouragement and a long lost hope of employment. What the findings further highlight is the value in running parallel courses of treatment and job support, as opposed to sequential courses of treatment first, then work rehabilitation. Apart from the drop-out, none of the participants thought that IPS interfered with their pain treatment. Rather, the continuous focus on work throughout the treatment contributed to a mind shift where work was considered part of the pain rehabilitation process instead of just the end point of a successful treatment. Both these aspects are essential parts of IPS [24], and suggest that this might be a good model for patients with chronic pain and no employment.

Most participants in this study emphasized that work was important for both physical and mental well-being. They explained that work was associated with feeling useful, having a good self-esteem, building hope and a feeling of moving forward. Having the opportunity to work, and as such be “normal” were highlighted by the participants. This corresponds well with the widely documented benefits of work on both physical and mental health [32]. Participants did, however, mention several challenges as well. The challenges included lack of education, long-term work absence, the writing of applications, gaps in the CV, pain, fear of setbacks and the short duration of the job support. These challenges correspond well with previously reported barriers to employment in patients with chronic pain. For example, Busch et al. [33] found that long-term work absence predicted sustained long-term sick leave in people with chronic musculoskeletal pain. Pain and fear that work might increase pain or cause harm are also reported as risk factors for chronic work disability [34, 35]. Moreover, lower levels of education are associated with higher functional disability [36] and lower employment rates [37] in patients with chronic pain conditions.

No participants reported unhelpful elements of the IPS. However, one participant dropped out early from the study due to exacerbation of symptoms. An external physical therapist advised this participant to withdraw from the study because she believed that IPS interfered with the treatment. The treatment team at the pain clinic on the other hand, disagreed with the external therapist’s advice. Indeed, an increased activity level, changes in the daily routine, cognitive challenges, and going through stressful situations such as job-interviews may worsen the pain (e.g. [38]). However, participants in this study stressed that work also shifted their focus away from the pain. There is no evidence suggesting that IPS should be discouraged in the beginning of a new treatment program or in a period of symptom exacerbation. In our opinion, tolerable and harmless changes or exacerbations of symptoms should not be considered as contraindications for the participant to engage in work related activities. The conflicting perspectives of the external therapist and the pain clinic team in the current study, highlight the importance of “all players on the same side” in pain rehabilitation. Importantly, good communication between different therapists, as well as a close and integrated treatment plan, seem to be advisable.

To the best of our knowledge, this is the first study investigating IPS in patients struggling with chronic pain. IPS was originally designed for people with mental illness. Nevertheless, recent initiatives have attempted to expand the IPS methodology to clients with other conditions, including veterans with post-traumatic stress disorder [39] and spinal cord injuries [40]. Despite some similarities between the latter group and the current study population, chronic pain patients are still a distinct and much more prevalent group. Work absence and social benefits for this patient group alone constitutes a tremendous economical expense for the society, as well as economical and psychosocial expenses for the individual patient and their families [4]. In our opinion, having a pain condition does not contradict engagement in work related activities. However, as pointed out by our study participants, returning to work after a period of illness can be challenging. There is thus a great need for effective work rehabilitation models for patients with chronic pain. Numerous attempts have been made to help patients with various pain conditions to return to work, but with modest results [41], except for a few successful examples [42, 43].

In this study, the employment status changed for three participants. The employment rate of 38% over a 12 months period appears promising. It is slightly better than employment rates in patients with mental disorders who participated in regular return-to-work programs (7–40%), but lower than the employment rates in patients with mental disorders who participated in IPS programs (22–78%) [14]. There may be several reasons for the lower employment rate in the current study. First, the study sample was small (*n* = 8) which makes the employment rate highly susceptible to sampling variation. Second, the 3 months scheduled for the IPS intervention was not sufficient time for the employment specialists to find a job match. Time and economical restrictions were the reasons for the short timeframe of the IPS intervention in the present study. However, three participants received an extended intervention and follow-up. Two of these participants were employed after 6 and 12 months, suggesting that there is a need to follow the participants for more than 3 months. In fact, most IPS studies report the employment-rate after 6–12 months of intervention and follow-up. Lastly, patients with chronic pain may face other and more debilitating challenges with job support and employment than other patient groups. For example, fear avoidance, which refers to the avoidance of movements or activities based on fear of exacerbation of symptoms, is found to be related to disability and work loss [44–46]. Waddell et al. [45] reported that fear-avoidance beliefs about physical activities and work were strongly related to disability and work loss in the previous year, and even more so than biomedical variables and characteristics of pain. In the current study, no obvious patterns emerged among the participants’ pain condition, disability level, and how well they responded to the intervention. A correlation among these variables should be investigated in future studies with a larger sample size in order to identify the best candidates for IPS among the large group of patients with chronic pain.

Note that this study was never designed to establish the employment rate with IPS in chronic pain patients. Employment-rate and predictors of employment status after IPS intervention will be investigated in an on-going large randomized controlled trial on IPS in patients with chronic pain.

### Limitations

The pilot study has several limitations. First, the number of participants was low. Given the qualitative nature of the study, however, transferability was considered more important than generalizability, and data saturation superior to sample size. The study context was thus thoroughly described to ensure transferability, and the richness of the data critically reviewed to ensure saturation. Nevertheless, follow-up studies with larger samples are obviously warranted. Second, actual follow-up time in the study was very short for some participants (see Table 2), and the duration of employment support was similarly short. The participants also emphasized the short time-frame as a major challenge. Third, the time of year affected the study in the sense that two of the participants went abroad on holiday almost immediately after inclusion and for the remaining time of the study. Yet another participant wanted to postpone job start until after surgery, making it virtually impossible to offer IPS services from the time of inclusion and for the remaining time of the study. This again resulted in a limited period of job support. Most IPS studies have demonstrated that a longer duration is needed in order to obtain employment results, which was also evident in the study’s employment results - especially considering the participants in the extended follow-up group who did obtain employment. We would still argue, though, that 3 months is sufficient when the goal is to assess feasibility of the IPS model for a new target group and provide a rational for a randomized controlled trial. Another limitation of the study is the lack of self-report measures after IPS was delivered. Then again, the objective of the study was to investigate participants’ experiences with IPS, as they are expressed in interviews and not on self-report questionnaires.

Finally, whether data saturation was really obtained could always be questioned. The inclusion of more participants might have revealed additional relevant experiences besides from what is reported here. We would still argue though that the study has generated new and relevant knowledge of chronic pain patients’ experiences with the IPS model. Both the experiences and the employment outcomes suggest the potential of re-purposing IPS to chronic pain, which should be followed up and tested in larger studies with rigorous designs.

## Conclusion

This pilot study is the first report of the IPS model of supported employment applied in an outpatient setting for chronic pain patients. The results suggest that IPS is a feasible model to integrate with interdisciplinary pain rehabilitation, and should thus be tested out large scale in a randomized controlled trial.

## Abbreviations

EQ-5D: European quality of life**-**5 dimensions
HSCL-25: The Hopkins symptom checklist
IPS: Individual placement and support
NOK: Norwegian kroners
ODI: Oswestry disability index
STC: Systematic text condensation

## Footnotes

1 We used a modified version of the Oswestry Disability Index (ODI), as we removed the word “back” in the introduction part of the questionnaire.
